# High-Throughput 3D Bioprinted Organoids of Skin Cancer Utilized for Diagnosis and Personalized Therapy

**DOI:** 10.3390/curroncol32120653

**Published:** 2025-11-21

**Authors:** Arvind Kumar Shukla, Sandhya Shukla, Sonali Pradeep Suryawanshi, Adarsha Mahendra Upadhyay, Navin Ray, Govindhan Thiruppathi, Sayan Deb Dutta, Raj Kumar Mongre

**Affiliations:** 1 School of Biomedical Convergence Engineering, Pusan National University, Yangsan 50612, Republic of Korea; 2 Department of Pharmacology, Bharti Vidyapeeth Deemed University Medical College, Pune 411043, India; sandhyashukla020@gmail.com (S.S.); suryawanshi.sonali@bharatividyapeeth.edu (S.P.S.); 3 Department of Gastrointestinal Surgery, School of Overseas Education, Guizhou Medical University, Guiyang 550025, China; adarshupadhyay595@gmail.com; 4 Laboratory of Mucosal Exposome and Biomodulation, Department of Integrative Biomedical Sciences, Pusan National University, Yangsan 50612, Republic of Korea; navin.ray@gmail.com (N.R.); thiruppathigovindhan555@gmail.com (G.T.); 5 Department of Biosystems Engineering, Kangwon National University, Chuncheon 24341, Republic of Korea; sayan91dutta@gmail.com; 6 Institute of Forest Science, Kangwon National University, Chuncheon 24341, Republic of Korea; 7 School of Medicine, University of California, Davis, Sacramento, CA 95817, USA; 8 Department of Surgery, Boston Children’s Hospital, Harvard Medical School, Harvard University, 300 Longwood Ave, Boston, MA 02115, USA

**Keywords:** 3D bioprinting, skin cancer organoids, high-throughput screening, personalized therapy, tumor microenvironment, precision oncology

## Abstract

Recent advancements in three-dimensional (3D) bioprinting have revolutionized the modeling of skin cancer, enabling the fabrication of high-throughput, patient-specific organoids that recapitulate the structural, functional, and microenvironmental complexity of native tumors. This review focuses on the integration of cutting-edge bioprinting technologies with bioengineered extracellular matrices and patient-derived cells to generate physiologically relevant skin cancer models for diagnostic and personalized medicine applications. Key technological innovations, including novel bioinks, multi-material printing strategies, and biomimetic approaches, are discussed for their ability to replicate tumor-stroma interactions, vascularization, and immune microenvironments. The utility of bioprinted organoids in high-throughput drug screening, mutation-targeted therapy design, and biomarker discovery is critically evaluated. Additionally, we address current challenges in standardization, reproducibility, and clinical translation, highlighting regulatory and quality-control considerations. Collectively, this review emphasizes the transformative potential of 3D bioprinted skin cancer organoids as platforms for precision oncology, bridging bioengineering advances with translational research to accelerate therapeutic development and personalized treatment strategies.

## Simple Summary

Skin cancer is a serious global health issue that requires better models to study disease progression and treatment responses. Advances in 3D bioprinting now allow the creation of patient-specific skin cancer organoids that closely resemble real tumors. This review discusses how 3D bioprinting, combined with bioengineered materials and patient cells, can produce realistic tumor models for drug testing and personalized medicine. It also highlights key bioinks, printing methods, and strategies to mimic the tumor environment, along with challenges and future directions. Overall, 3D bioprinted organoids hold great promise for improving skin cancer research and precision therapy.

## 1. Introduction

The most common cancer in the world, skin cancer, includes a wide range of neoplastic conditions that originate from different types of skin cells. The three main types are melanoma, squamous cell carcinoma (SCC), and basal cell carcinoma (BCC). Although melanoma is less common, it accounts for the majority of skin cancer-related deaths. Different cellular lineages give rise to these cancers: melanocytes cause melanoma, squamous cells cause SCC, and basal keratinocytes cause BCC [1,2,3]. Melanoma is particularly aggressive among them because of its high potential for metastasis, intricate mutational landscape, and ability to evade the immune system. Skin cancer has a complex etiology that includes both environmental and genetic factors. The most important risk factor is still ultraviolet (UV) radiation from sunshine, which can cause DNA damage, mutations in tumor suppressor genes like TP53, and the activation of oncogenes like BRAF, NRAS, and CDKN2A [4,5,6]. Tumor initiation and progression are also influenced by immunosuppression, chronic inflammation, and carcinogen exposure. Cancer cell survival, drug resistance, and immune escape are all significantly aided by the tumor microenvironment (TME), which is made up of stromal cells, immune cells, extracellular matrix (ECM), and signaling molecules. The genetic, phenotypic, and epigenetic diversity of skin cancers has been revealed by recent developments in genomics and molecular biology. Effective diagnosis and treatment are severely hampered by this complexity. Although targeted treatments (e.g., G. immune checkpoint inhibitors (e.g., BRAF and MEK inhibitors) [7,8,9]. Although anti-PD-1/PD-L1 treatments have greatly improved outcomes for patients with metastatic melanoma, many still experience resistance or relapse. Therefore, to screen for customized treatment regimens and gain a better understanding of tumor behavior, it is imperative to develop trustworthy, patient-representative preclinical models [10,11,12,13,14].

**Figure 1:**
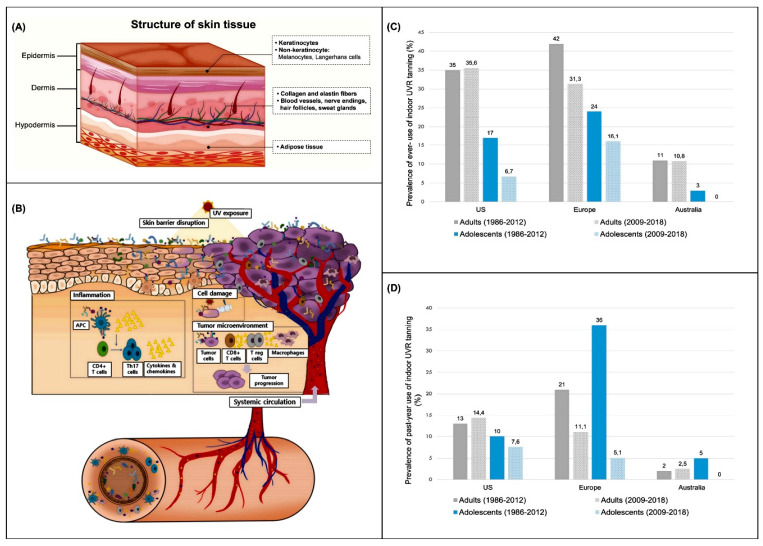
Structural and mechanistic insights into skin tissue and UV-induced skin cancer development. (**A**) Illustration of the anatomical structure of skin tissue, depicting its key layers and cellular components involved in maintaining skin integrity and function [15]. (**B**) Conceptual framework outlining how ultraviolet (UV) exposure contributes to skin cancer through disruption of the skin microbiome and skin–gut axis. UV radiation compromises the skin barrier and alters the balance of commensal skin microbes. These changes, along with the release of damage-associated molecular patterns (DAMPs), pathogen-associated molecular patterns (PAMPs), and microbial toxins, can lead to persistent inflammation and DNA damage. These pathological events promote tumor initiation and progression. Immune cells—such as cytotoxic CD8+ T cells, regulatory T cells, and tumor-associated macrophages—alongside their secreted cytokines and chemokines, play crucial roles in shaping the immunosuppressive and pro-inflammatory tumor microenvironment. Additionally, microbial metabolites, inflammatory mediators, and signaling molecules originating from the gut microbiota can circulate systemically, influencing skin tumor development and progression from a distance, highlighting the interplay between the gut and skin in cancer pathogenesis [16]. (**C**) Aggregated data from published meta-analyses presenting the percentage of individuals who have ever used indoor ultraviolet radiation (UVR) tanning devices, reflecting lifetime exposure trends [17]. (**D**) Compiled prevalence rates from meta-analyses reporting past-year usage of indoor UVR tanning, emphasizing recent behavioral patterns related to artificial UV exposure and associated cancer risks [17]. This figure is adapted from Refs. [15,16,17] under the Creative Commons Attribution 4.0 (CC BY 4.0) license.

The skin is the largest and most physiologically diverse organ in the human body and is responsible for barrier protection, immune modulation, thermoregulation, and sensory recognition. Its unique, multilayered structure is directly related to these critical functions, as shown in Figure 1A [15]. The skin is composed of three main layers: the epidermis, which contains proliferative basal keratinocytes, melanocytes that are involved in pigmentation and UV defense, and antigen-presenting Langerhans cells, the dermis, which provides structural support and elasticity through collagen and elastin and houses blood vessels, nerve endings, and adnexal structures, and the hypodermis, which contains adipose tissue that is necessary for energy storage, insulation, and mechanical cushioning. Skin cancer is caused by the malignant transformation of these components, often caused by ultraviolet (UV) radiation. The three most common types of skin cancer are basal cell carcinoma (BCC), squamous cell carcinoma (SCC), and melanoma. The most common and least active form of BCC is basal keratinocytes, which are derived from the basal keratinocytes. SCC is more superficial and more likely to cause metastasis.

Melanoma is the most lethal form of melanocyte, and it is characterized by its aggressive metastatic behavior and immune evasion capabilities. Merkel cell carcinoma, cutaneous lymphoma, and Kaposi’s sarcoma are examples of rare but significant skin malignancies, and underscore the role of immunosuppression and viral co-infection in skin carcinogenesis. The skin microbiota and gut–skin axis are now being used to promote skin cancers, as shown in Figure 1B [16]. UV-induced barrier disruption alters the commensal microbial balance, causing chronic inflammation through the release of pathogen- and damage-associated molecular patterns (PAMPs and DAMPs) and microbial toxins. These factors influence immune cell behavior (CD8+ T cells, regulatory T cells, and tumor-associated macrophages) within the tumor microenvironment, promoting immune suppression, proliferation, angiogenesis, and tumor progression. Additionally, gut-derived metabolites and cytokines can enter systemic circulation and modulate skin immunity, indicating a complex interaction between internal and external environments in cancer development [16]. Figure 1C,D indicate that indoor UVR tanning is a common risk factor for non-melanoma and melanoma skin cancers worldwide, and that indoor UVR tanning is a common risk factor for non-melanoma and melanoma skin cancers [17,18,19]. Wehner et al., A meta-analysis. The lifetime (“ever-use”) prevalence of indoor tanning ranged from 11% in Australia to 42% in Europe (Ross, 1986–2012). Past-year prevalence ranged from 2% to 21%, with adolescents using more than adults. A meta-analysis of Rodriguez-Acevedo et al. In 2009–2018, after the WHO classified indoor tanning as a Group 1 carcinogen, usage decreased, but gender disparity continued. Females were more likely than males to tan than females (16.8% vs. 8.5%), and adolescent girls were more likely than boys to tan (8.9% vs. 3.9%). These patterns, as shown in Figure 1C,D, illustrate the ongoing public health challenge of artificial UVR exposure and its significant contribution to the global skin cancer burden [17,18,19,20].

Traditional in vitro and in vivo models are essential for comprehending cancer biology, but they have major drawbacks when it comes to translational research in skin cancer. Due to their affordability and ease of use, two-dimensional (2D) monolayer cultures of cancer cell lines are now widely used [21,22,23]. The cellular heterogeneity, dynamic interactions with the TME, and three-dimensional architecture seen in vivo are not, however, replicated by them. Their inability to model tumor-stroma and tumor-immune cell crosstalk limits their ability to predict drug response. Patient-derived xenografts (PDXs) and genetically engineered mouse models (GEMMs) are two examples of animal models that can replicate some aspects of tumor progression and metastasis and provide a more complex biological context [24,25,26]. Nevertheless, their translational value is limited by interspecies immune system differences, ethical considerations, lengthy generation times, and high maintenance costs. Furthermore, these models frequently do not permit individualized evaluations of therapeutic efficacy or high-throughput screening. Furthermore, traditional organoid cultures lack the precise spatial organization and vascularization required to fully model the complexity of skin cancer, even though they are more physiologically relevant than 2D models. Active ECM embedding is a prerequisite for standard organoid protocols (e.g., 3. Heterogeneity and irregular morphology could be the outcome of spontaneous self-assembly and Matrigel. Significant obstacles to scalability, reproducibility, and integration with high-throughput drug testing platforms are presented by these limitations [27,28,29,30].

Three-dimensional (3D) bioprinting has emerged as a transformative technology in cancer research, offering unparalleled precision in replicating the complex architecture and cellular heterogeneity of native tissues. In the context of skin cancer, 3D bioprinting enables the fabrication of physiologically relevant tumor models that closely mimic the structural, biochemical, and mechanical properties of the native tumor microenvironment [31,32,33]. By integrating patient-derived cells, extracellular matrix components, and spatial organization, these bioprinted constructs facilitate a more accurate understanding of tumor progression, invasion, and metastasis. Moreover, such models serve as powerful platforms for high-throughput therapeutic screening, enabling personalized and predictive drug testing while reducing reliance on traditional two-dimensional cultures and animal models [34,35]. The human skin, a multilayered organ composed of the epidermis, dermis, and hypodermis, plays a critical role in maintaining barrier function and homeostasis. Tumorigenic transformation within the skin disrupts this architecture, leading to alterations in cellular communication, extracellular matrix remodeling, and immune cell infiltration. The skin tumor microenvironment is highly dynamic, characterized by reciprocal interactions among keratinocytes, fibroblasts, endothelial cells, and immune components that collectively regulate cancer initiation and progression. Understanding these microenvironmental dynamics through advanced 3D bioprinted skin cancer models provides valuable insights into tumor biology and paves the way for developing effective and targeted therapeutic strategies [36].

Three dimensions (3D) Bioprinting has become a revolutionary method for creating intricate, multicellular, and spatially ordered tissue models. The use of biofabrication technologies in 3D bioprinting allows for the accurate deposition of various cell types, biomaterials, and signaling factors in predetermined architectures that replicate the structure and function of native tissue. An unparalleled chance to accurately and precisely replicate the tumor microenvironment in vitro is presented by this technology. Organoids that closely mimic the layered structure of human skin, including the epidermis, dermis, and underlying vasculature, can be produced using 3D bioprinting in the context of skin cancer. To replicate the complexity of the TME, bioprinted skin cancer organoids can include immune components, fibroblasts, keratinocytes, melanocytes, endothelial cells, and cancer cells [37,38,39]. When assessing immune responses, drug resistance mechanisms, and tumor-stroma interactions in patient-specific settings, this high level of biomimicry is essential. Additionally, smaller organoid arrays that are appropriate for multiplexed drug screening can be produced quickly and reliably using high-throughput bioprinting platforms. These systems can be combined with imaging, omics-based readouts, and automated liquid handling to provide thorough information on drug toxicity, efficacy, and molecular response signatures. Crucially, patient-derived tumor cells or biopsy samples can be used to create customized tumor avatars that act as ex vivo stand-ins for clinical judgment [40,41,42,43]. From a medical standpoint, bioprinted skin cancer organoids are an effective precision oncology tool. They may be used to reduce the trial-and-error method that predominates in contemporary clinical practice by screening for the best treatment plans on an individual basis before starting therapy. By better capturing the diversity and progression of skin tumors than conventional systems, these models can also speed up the discovery of new therapeutic targets and biomarkers. Bioprinting technologies are expected to become increasingly useful in cancer research as they develop further, thanks to developments in bioinks, crosslinking chemistries, and microfluidic integration. High-throughput screening platforms, patient-derived organoid culture, and 3D bioprinting come together to usher in a new era of functional diagnostics and tailored treatment in dermatologic oncology [31,39,44].

The convergence of three-dimensional (3D) bioprinting technologies with biological modeling represents a transformative approach in skin cancer research. Recent advances in biofabrication enable the generation of patient-specific organoids that faithfully recapitulate the structural and functional complexity of native tumors, including their microenvironmental heterogeneity [45]. By integrating cellular, extracellular, and mechanical cues within a precisely engineered 3D matrix, these models overcome many limitations of conventional two-dimensional cultures, providing more physiologically relevant platforms for studying tumor biology [46,47]. This review highlights how 3D bioprinting methodologies, including scaffold-based and scaffold-free approaches, support the fabrication of reproducible and high-throughput skin cancer organoids, thereby facilitating mechanistic investigations at both cellular and molecular levels. Beyond methodological considerations, the central focus of this work lies in the translational potential of bioprinted skin cancer organoids. These models not only enable systematic drug screening and evaluation of therapeutic responses but also provide a foundation for precision oncology by allowing patient-specific predictions of treatment efficacy. By bridging cutting-edge bioengineering strategies with clinical applications, this review provides a cohesive framework that underscores the promise of 3D bioprinting as a tool for advancing mechanistic understanding, personalized therapy, and future clinical decision-making in skin cancer management.

In this review, we discuss the recent advancements in the development and application of high-throughput 3D bioprinted organoids for modeling skin cancer. A discussion of the biology of skin cancer is given first, along with a discussion of the shortcomings of traditional 2D and in vivo models. Future developments in 3D bioprinting, bioink technology, and methods for creating biomimetic skin cancer organoids are the main topics of the review. We also investigate how to incorporate these models into high-throughput platforms for molecular profiling, drug screening, and customized treatment. Lastly, we assess the field’s present difficulties, clinical translation prospects, and potential future paths in precision dermatologic oncology. In conclusion, the development of complex, patient-representative platforms is required due to the complexity of skin cancer biology and the limitations of traditional preclinical models. A promising method for accurately simulating the cellular, molecular, and structural characteristics of skin tumors is the use of high-throughput 3D bioprinted organoids. In addition to improving our knowledge of the pathophysiology of skin cancer, these biomimetic systems provide reliable platforms for immunotherapy assessment and individualized medication screening. The combination of bioprinting and clinical and molecular oncology has enormous potential to revolutionize the diagnosis and treatment of skin cancer as the field develops.

## 2. Advances in Skin Cancer Modeling

Skin cancer, which can be broadly classified into melanoma and non-melanoma skin cancers (NMSCs), which include squamous cell carcinoma (SCC) and basal cell carcinoma (BCC), is one of the most common cancers in the world. The majority of skin cancer-related deaths are caused by melanoma, which is derived from melanocytes and is much more aggressive than NMSCs despite being less common [3,48,49]. The most prevalent type of cancer, basal keratinocyte-derived BCC, usually grows slowly, whereas squamous keratinocyte-derived SCC has a higher potential for metastasis than BCC. Genetic mutations interact intricately to drive the development of skin cancer (e.g., A. PTCH1, CDKN2A, BRAF, TP53, and epigenetic modifications), as well as environmental elements like UV rays, persistent inflammation, and immunosuppression. To develop effective diagnostic and therapeutic strategies, it is essential to understand the multistep process of skin carcinogenesis, which encompasses normal skin epithelium, dysplasia, carcinoma in situ, and invasive carcinoma [32,50,51,52].

Three-dimensional (3D) bioprinting has emerged as a transformative technology in tissue engineering and disease modeling, which provide precision in recreating complex tissue architectures. By depositing living cells, biomaterials, and bioactive molecules layer-by-layer, 3D bioprinting enables the fabrication of physiologically relevant tissue constructs for both in vitro and in vivo applications [53]. Recent advancements have focused on integrating biomimetic matrices, such as extracellular matrix (ECM)-derived hydrogels, with immune microenvironmental elements to recapitulate the dynamic crosstalk between tumor cells, stromal components, and immune populations. This integration enhances cellular viability, promotes functional tissue maturation, and allows for the interrogation of tumor–immune interactions under controlled conditions [54]. Different laboratories have explored combinations of natural and synthetic matrices, crosslinking strategies, and immune modulatory cues to optimize construct fidelity and experimental reproducibility [54,55,56]. Building upon these technological foundations, 3D bioprinted models have been applied in vitro to study disease-specific mechanisms and screen therapeutic responses, and in vivo for tissue regeneration and preclinical disease modeling. By centralizing the bioprinting approach and systematically integrating matrix and immune components, researchers can generate high-fidelity models of complex pathologies, enabling mechanistic studies, high-throughput drug screening, and personalized therapy testing, thus demonstrating the translational potential of 3D bioprinting in regenerative medicine and oncology [57,58].

**Figure 2:**
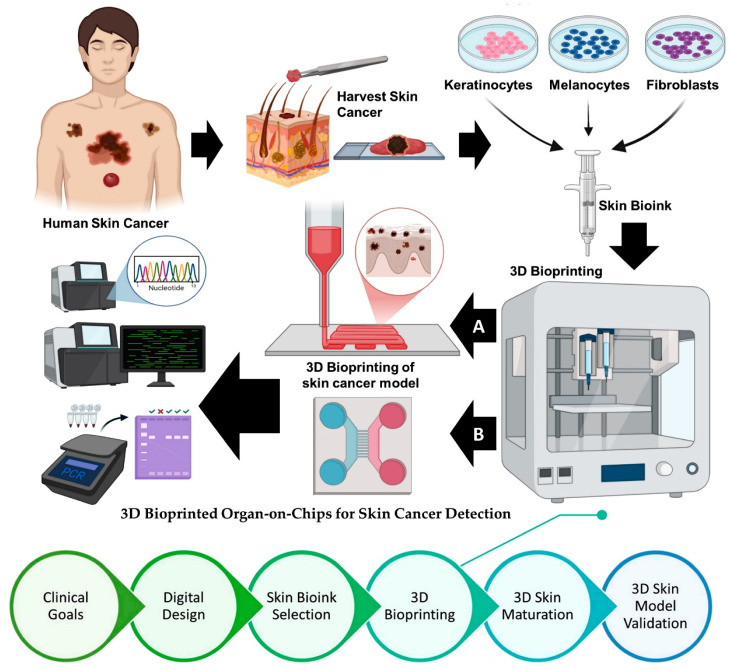
The process of 3D bioprinting human-equivalent skin cancer models using organ-on-chips for skin cancer detection. (A) 3D bioprinting of a skin cancer model. (B) 3D bioprinted organ-on-chips for skin cancer detection. Created with BioRender.com (accessed on 25 July 2025).

The skin bioprinting process is divided into six main steps, as shown in Figure 2. The first step is determining clinical goals, in which the researcher sets research and clinical objectives following a project design criterion based on diseases, real-world structures to imitate, and pre-existing models. To create 3D digital models and create tool paths, respectively, the second step involves using 3D computer-aided design software and computer-aided manufacturing software. Next, choosing a bioink is a crucial step in the bioprinting process. To choose the best bioink, background information on the material properties of the selected bioink, as well as the physiological and biochemical conditions of the cells, is needed. A 3D bioprinter, printheads, temperature control, and sterile conditions are necessary for the fourth step, which is the printing process. In order to achieve high resolution, this step includes an optimization sub-step that is primarily used for parameter adjustment. Functionalization is the fifth step, in which the printed model is put into a bioreactor or incubator to enable the cells to grow, stabilize, and become functional. The model’s suitability for its intended use is verified in the sixth and last step. In vitro testing, disease modeling, and in vivo implementation are common uses.

Skin cancer is traditionally studied using xenografts, genetically engineered mouse models (GEMMs), 2D cell lines, and patient-derived xenografts (PDXs). Although 2D monolayer cultures are inexpensive and simple to manage, they lack the cellular heterogeneity, microenvironmental context, and three-dimensional architecture of in vivo tumors [59,60,61]. Their predictive ability for drug screening and comprehending disease mechanisms is thus limited. In contrast, animal models, particularly mouse models, provide systems that are more physiologically relevant by taking into account systemic effects and immune interactions. GEMMs, like those with conditional Tp53 or BrafV600E mutations, mimic some features of the onset and spread of human skin cancer [62,63,64]. Tumor fragments from patients are implanted into immunocompromised mice in PDXs, which have proven useful in assessing treatment outcomes. However, due to ethical concerns and species-specific variations, these in vivo systems are costly, time-consuming, and limited. Three-dimensional (3D) culture models, such as organoids, scaffolds, and spheroids, have become popular for bridging the gap between in vivo and in vitro research. For high-throughput drug screening and personalized medicine applications, organoids—in particular—are perfect because they can more closely resemble the cellular complexity and architecture of tumors. These models still have issues with reproducibility, standardization, and integrating various skin constituents like immune cells and vasculature [65,66,67].

3D bioprinted systems can recapitulate key components of the tumor microenvironment, including stromal and immune interactions, by incorporating cancer-associated fibroblasts (CAFs), macrophages, and T cells within biomimetic extracellular matrices. CAFs actively remodel the matrix, influencing tumor stiffness and drug penetration, while macrophage polarization (M1 vs. M2) modulates inflammatory and immunosuppressive cues. Additionally, bioprinted architectures allow controlled T-cell infiltration, enabling evaluation of immune-mediated cytotoxicity. Collectively, these systems provide a physiologically relevant platform to assess how stromal and immune dynamics affect therapeutic responses [68,69]. Immune evasion, treatment resistance, and the advancement of skin cancer are all significantly influenced by the tumor microenvironment (TME). It is made up of a dynamic and interactive network of immune cells (e.g., CAFs), cancer-associated fibroblasts (e.g., T cells, macrophages), endothelial cells, signaling molecules, and extracellular matrix (ECM) constituents. Both innate and acquired resistance to targeted treatments, including BRAF inhibitors and immune checkpoint blockade, are greatly influenced by the TME in melanoma (e.g., A. anti-PD-1/PD-L1 treatments [70,71,72]. Heterogeneity and plasticity are two of the TME’s primary characteristics, allowing cancer cells to adjust and endure under therapeutic pressure. CAFs can secrete cytokines and remodel the extracellular matrix (e.g., A. that encourage angiogenesis, tumor growth, and drug resistance (e.g., TGF-β, IL-6). Immune checkpoint expression, myeloid-derived suppressor cells (MDSCs), and regulatory T cells (Tregs) all contribute to immune suppression in the TME, which considerably reduces the effectiveness of immunotherapies. Hypoxia in the TME also causes metabolic reprogramming and the epithelial-mesenchymal transition (EMT), both of which increase the aggressiveness of tumors. The complexity of the TME is not adequately captured by the majority of current models, despite these revelations. Improvements in organ-on-chip platforms, 3D bioprinting, and co-culture systems have made it possible to integrate TME components more effectively, increasing model fidelity and clinical relevance [73,74,75,76].

Our comprehension of tumor biology and treatment response is being revolutionized by recent developments in skin cancer modeling. The intricate spatial and cellular architecture of human tumors cannot be accurately replicated by conventional 2D cultures and animal models, despite the fact that they have yielded insightful information. New 3D models that incorporate tumor heterogeneity and microenvironmental cues are particularly promising, especially patient-derived organoids and bioengineered constructs. It is crucial to include elements of the TME in these models in order to investigate therapy resistance mechanisms and find new therapeutic targets. As these models develop further, they have the potential to significantly improve outcomes for skin cancer patients, speed up drug development, and improve personalized medicine.

## 3. Bioprinting Technologies for Skin Cancer Organoids

The development of biomimetic models for the study of complex diseases like skin cancer has advanced dramatically with the advent of 3D bioprinting. The complex architecture, heterogeneity, and microenvironmental dynamics of human skin tumors cannot be replicated by conventional 2D culture systems. On the other hand, skin cancer organoids that closely resemble native tissues can be created thanks to 3D bioprinting, which provides precise spatial control over a variety of cell types and extracellular matrices (ECMs). These organoids are excellent tools for researching immunological evasion, drug resistance, tumorigenesis, and creating individualized treatment plans [77,78,79]. A number of 3D bioprinting technologies have been created to create tissue-mimicking structures that are accurate, quick, and scalable. Extrusion-based bioprinting, laser-assisted bioprinting, and inkjet bioprinting are the three main bioprinting methods. Bioinks are deposited in droplet form using thermal or piezoelectric forces in inkjet bioprinting. Although the high speed and cost-effectiveness of this method are beneficial, the formation of densely populated tumor regions may be impeded by its low viscosity requirements and decreased cell density.

**Figure 3:**
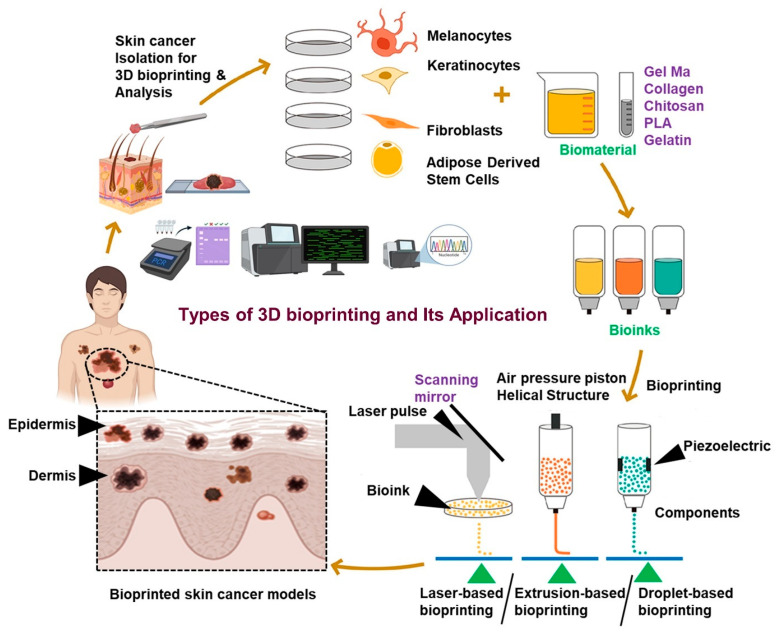
Types of 3D bioprinting and their applications for the fabrication of skin cancer models for the detection and analysis of cancer and the potential effects of various drugs. Created with BioRender.com (accessed on 25 July 2025).

The most popular method for creating cancer organoids is extrusion-based bioprinting, which extrudes bioinks continuously through a nozzle using mechanical or pneumatic forces. It enables the creation of layered structures with high mechanical stability and cell viability by printing dense cell suspensions and high-viscosity materials [80,81,82]. The stratified architecture of skin, including the epidermis, dermis, and tumor mass, can be modeled with this technique. Laser-assisted bioprinting uses a concentrated laser to drive bioink droplets from a donor slide to a collector surface, providing high resolution and cell viability. Although it avoids nozzle clogging and offers precision, its cost and complexity may prevent it from being widely used. In general, the platform selection is based on the intended resolution, cell viability, biological objective, and compatibility with particular bioinks with specific types of 3D bioprinting techniques utilized for the development of skin cancer models and its application for detection, analysis of cancer, and the potential effects of various drugs, as shown in Figure 3.

Bioinks are essential for bioprinting skin cancer organoids because they affect tumor microenvironmental interactions, differentiation, and cell viability. The best bioinks for skin cancer modeling should support cell adhesion, permit vascularization and tumor growth, and replicate the characteristics of the natural extracellular matrix. Natural bioinks provide biological cues and biocompatibility that are vital for the growth of skin and tumor cells. Examples of these include collagen, gelatin, hyaluronic acid, alginate, and decellularized extracellular matrix (dECM). As an illustration, blends of alginate and gelatin-methacryloyl (GelMA) enable adjustable mechanical characteristics and promote the growth of melanoma and keratinocyte cells. While Pluronic F127 and polyethylene glycol (PEG) derivatives are examples of synthetic bioinks that provide fine control over mechanical and chemical properties, they frequently lack bioactivity. Hybrid bioinks that combine natural and synthetic polymers have been created to address this issue and combine their benefits. Methods of crosslinking, like ionic (e.g., heat, calcium ions for alginate gelatin), photocrosslinking (e.g., Enzymatic techniques and UV light for GelMA), are used to stabilize the printed structures. Because photo-crosslinking gels quickly and allow for spatial control, it is particularly well-liked for high-throughput applications. A comparative analysis of bioprinting methods for skin cancer models, including advantages and disadvantages, is presented in Table 1 [82,83,84].

To create skin cancer organoids that mimic the intricacy of natural tissue, several cell types must be layered in precise spatial configurations. To replicate the epidermal, dermal, and vascular compartments, a biomimetic skin cancer model usually consists of basal keratinocytes, melanocytes, fibroblasts, endothelial cells, and immune cells. Using keratinocytes and melanoma cells to create the epidermis and fibroblasts embedded in a collagen-rich matrix to create the dermis, bioprinting allows for the creation of multi-layered structures. Integrating immune cells (e.g., cancer-associated fibroblasts, or CAFs) helps address tumor heterogeneity [85,86,87]. To replicate the varied cellular and molecular environment of skin tumors using patient-derived tumor cells, macrophages, T cells, and others. Furthermore, the ability to replicate immune infiltration and nutrient diffusion depends on vascularization. Techniques like sacrificial ink removal and coaxial bioprinting enable the development of perfusable vascular channels inside the constructs. Through the introduction of fluid flow, oxygen gradients, and dynamic drug exposure conditions that replicate the in vivo tumor microenvironment, integration with microfluidic systems further improves the physiological relevance [88,89,90].

Various 3D bioprinting platforms offer distinct capabilities for modeling skin cancer, each with unique advantages and limitations. Scaffold-based approaches provide structural support and precise spatial control but may introduce biomaterial-associated artifacts. Scaffold-free or spheroid-based methods better replicate native cell–cell interactions, though maintaining long-term tissue integrity can be challenging. Integration with microfluidic systems enables vascularization and dynamic modeling of the tumor microenvironment, supporting more physiologically relevant functional assays, albeit with increased technical complexity. These features collectively influence the translational potential of each platform for mechanistic studies, drug screening, and personalized therapy, as shown in Table 2.

**Table 1:** Comparative overview of bioprinting techniques for skin cancer models.

Bioprinting Technique	Specific Bioinks Used	Advantages	Disadvantages	Ref.
Laser-based Bioprinting (e.g., Laser-Assisted, DLW, LIFT)	- Gelatin methacrylate (GelMA) - Collagen - Cell-laden hydrogels - Photosensitive hydrogels	- High spatial resolution (micron-scale precision) - Minimal thermal or mechanical stress on cells - Enables fabrication of complex, multicellular structures, including microvasculature - Supports multi-material patterning	- Requires photosensitive bioinks - Low throughput for large-scale constructs - High equipment cost and operational complexity - Risk of photothermal damage - Limited bioink compatibility	[91,92,93]
Extrusion-based Bioprinting	- Collagen - Fibrin(ogen) - Gelatin - Hyaluronic acid - Alginate (with embedded cancer cells) - ECM-derived inks - Composite hydrogels	- Cost-effective and technically simple - Accommodates high - viscosity and high cell - density materials - Suitable for large, tissue-scale constructs - Compatible with diverse bioinks, both natural and synthetic	- Lower resolution (~100–200 μm) - Shear stress may affect cell viability - Soft constructs prone to collapse without support - Limited fidelity in recreating fine microarchitecture - Balance needed between printability and biofunctionality	[94,95,96]
Droplet-based Bioprinting (e.g., Inkjet, Microvalve, EHD Jetting)	- Collagen - Fibrin—PEG - based hydrogels—Plasma-derived matrices - Low-viscosity bioinks	- Allows precise droplet - based patterning - High cell viability due to non-contact deposition - Well-suited for multilayered, thin constructs - High reproducibility and low material waste	- Restricted to low-viscosity materials - Susceptible to nozzle clogging - Poor suitability for thick or volumetric structures - Lacks inherent porosity and mechanical robustness—Limited construct size and load-bearing capacity	[97,98,99]

**Table 2:** Comparative Analysis of 3D Bioprinting Platforms: Advantages, Limitations, and Translational Relevance.

Platform	Advantages	Limitations	Translational Relevance	Ref.
Scaffold-based 3D Bioprinting	Provides structural support for complex tissue architecture; allows precise spatial positioning of multiple cell types; compatible with various biomaterials	Limited by potential cytotoxicity of crosslinkers; may not fully mimic native tissue stiffness or ECM dynamics	Useful for modeling tumor-stroma interactions and testing drug penetration in structured tissue environments	[81,100,101,102]
Scaffold-free Bioprinting/Spheroid-based	Cells self-assemble into microtissues/organoids; avoids biomaterial-induced artifacts; closely mimics native cell–cell interactions	Challenges in maintaining long-term structural integrity; limited scalability	Effective for high-throughput drug screening and patient-specific organoid generation	[103,104]
Microfluidic-integrated Bioprinting	Enables perfusion, nutrient delivery, and vascularization; allows dynamic modeling of tumor microenvironment	Complex fabrication; requires specialized equipment; challenging to standardize	Supports functional assays of tumor growth, angiogenesis, and drug response under physiologically relevant flow	[105,106]

In conclusion, the development of organoids that faithfully replicate the structural and functional complexity of the tumor has been made possible by 3D bioprinting technologies, revolutionizing the field of skin cancer research. Robust and customized cancer models can be created by combining sophisticated bioprinting platforms, optimized bioinks, and engineered multicellular architectures. Advances in vascularization techniques, crosslinking chemistry, and biofabrication will improve the accuracy and translational significance of skin cancer organoids as the field develops. In the end, these developments will improve patient stratification, speed up drug discovery, and advance personalized oncology.

## 4. High-Throughput Strategies for Organoid Fabrication

High-throughput organoid fabrication techniques are essential for speeding up drug discovery and translational cancer research. In addition to being time-consuming and labor-intensive, traditional methods for creating cancer organoids frequently lack the scalability needed for extensive screening. These restrictions are being overcome by recent advancements in biofabrication technologies, specifically 3D bioprinting, microfluidics, and organ-on-chip systems, which make it possible to produce scalable, physiologically relevant, and reproducible skin cancer organoids. These high-throughput methods enable quick drug testing and genomic analysis, improve control over the tumor microenvironment, and provide precision in tissue architecture. With an emphasis on scalability and quality control, this section describes the creation of high-throughput organoid models via automation, multiplexed bioprinting, and the integration of microengineered systems [107,108,109].

Multiplexed and automated bioprinting platforms are transforming the production of skin cancer organoids. Clinical applications and drug discovery are limited by the high variability and low throughput of traditional manual organoid culture. Automated bioprinting systems, on the other hand, require little user intervention and can quickly deposit cells and biomaterials with spatial precision. From bioink loading to structure fabrication, these systems optimize the bioprinting process by leveraging digital control interfaces and programmable robotics. By creating several organoids with different compositions and geometries at once, multiplexed bioprinting improves throughput and experimental reproducibility. It is possible to deposit cell-rich hydrogels into multiwell formats or microarray platforms using methods like droplet-based printing and extrusion-based bioprinting. Under consistent conditions, this is especially beneficial for screening sizable drug panels or tumor cells derived from patients. Additionally, modern bioprinting systems incorporate feedback loops and real-time imaging, which enable accurate monitoring and correction throughout the printing process, lowering errors and variability in the production of organoids [34,110,111,112].

When combined with 3D bioprinted organoids, microfluidic systems, and organ-on-chip platforms offer a potent technological convergence that aims to replicate the physiology of native skin cancer. Microfluidic devices manipulate fluid flow, nutrient gradients, and chemical stimuli to provide precise control over the cellular microenvironment. By precisely delivering drug or immune effector concentrations in a time-dependent manner, these devices can be made to closely mimic in vivo pharmacodynamics. Because static cultures frequently lack dynamic conditions like shear stress, oxygen gradients, and immune infiltration, researchers can replicate these in the context of skin cancer through microfluidic integration. For example, skin cancer organoids can be used in tumor-on-a-chip platforms to mimic interactions between the tumor and the vasculature or the migration of metastatic cells across dermal barriers. Microfluidic perfusion also improves waste elimination and nutrient exchange, which prolongs culture viability and promotes the organoids’ functional maturation. Organ-on-chip technologies also make it easier to co-culture immune, stromal, and endothelial cells. This allows researchers to test immunotherapeutic approaches such as adoptive T cell therapy, immune checkpoint inhibitors, and others in a controlled microenvironment. These integrated systems enable the testing of patient-specific reactions to different therapeutic agents, making them useful tools for personalized medicine [113,114,115,116,117].

The effective use of organoid-based technologies in clinical and pharmaceutical applications depends on ensuring quality, scalability, and standardization. In high-throughput organoid fabrication, preserving consistency in size, cellular makeup, and structural integrity is one of the main obstacles. By precisely regulating printing parameters like extrusion speed, pressure, and temperature, automation helps to minimize batch-to-batch variability. To evaluate the quality of organoids in real time, sophisticated imaging techniques like live-cell imaging, optical coherence tomography, and confocal microscopy are being used. To confirm that organoids accurately replicate the phenotype and genotype of the original tumors, omics-based techniques such as transcriptomics and proteomics are employed. By using bioprinting platforms that work with common multiwell plates, scalability is accomplished (e.g., 96 or 384-well formats), allowing hundreds of organoids to be produced in parallel in a single run. Precision oncology applications and high-content drug screening depend on this. Additionally, to enhance inter-laboratory reproducibility and regulatory compliance, efforts are being made to create standardized protocols for the formulation of bioinks, the preparation of cell sources, and printing parameters [118,119,120,121].

**Figure 4:**
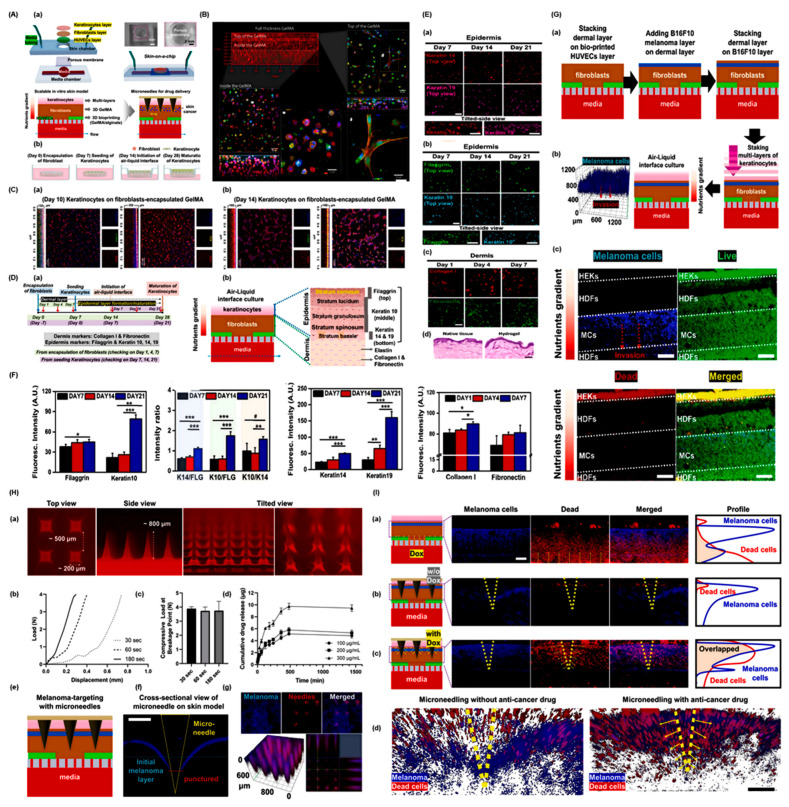
Comprehensive overview of the development, characterization, and therapeutic application of a 3D skin cancer-on-a-chip platform integrated with microneedles (MNs) for the detection and delivery of drugs. (**A**) Skin-on-a-chip setup and application strategy. (**a**) Schematic illustration of the 3D skin-on-a-chip setup, showing the PDMS chip, porous membrane, and skin-media chamber interface. Cross-sectional schematic views depict the skin model architecture and microneedle (MN) application for targeted drug delivery. (**b**) Air-liquid interface (ALI) culture method enabling stratified epidermal formation by keratinocyte differentiation. (**B**) ALI-cultured epidermal layers and cellular morphology. Confocal imaging reveals the 3D dermal structure. (**C**) Epidermal stratification and maturation. Confocal images of epidermal layers on days 10 (**a**) and 14 (**b**), showing nuclei (blue), actin (red), α-SMA (green), and pan-cytokeratin (yellow). (**D**) Structural and temporal development of skin layers. (**a**) Timeline of layered skin-on-a-chip formation. (**b**) Quantification of epidermal and dermal layer thicknesses. (**E**) Marker expression in skin layers. Confocal images and intensity profiles of (**a**) Keratin 14 and Keratin 19, (**b**) Filaggrin and Keratin 10, (**c**) Collagen I and Fibronectin. (**d**) Comparative H&E staining of native human skin and engineered skin tissue. Scale bars: 100 μm. (**F**) Spatial expression profiles of epidermal/dermal markers. Distribution and intensity of FLG (stratum corneum), K14 (stratum basale), and K10 (stratum lucidum–spinosum) across skin depths. (**G**) Skin cancer-on-a-chip model with metastatic melanoma. (**a**) Schematic showing melanoma cell layer incorporation. (**b**) Live/dead staining confirms melanoma invasion into the media channel after 24 h ALI culture. (**c**) 3D confocal image of melanoma within the model. Scale bars: 200 μm. (**H**) Characterization and drug release from DOX-loaded MNs. (a) Confocal images of MNs (tilted, side, and 3D views). (**b**) Stress-strain analysis, (**c**) compressive modulus with varied photo-crosslinking durations, (**d**) cumulative DOX release at varying concentrations. (**e**) Schematic of DOX-loaded MN application on skin cancer-on-a-chip. Representative cross-sectional images of MN insertion: (**f**) single needle and (**g**) MN array targeting the melanoma layer. Scale bars: 200 μm. (**I**) Transdermal DOX delivery and therapeutic response. Confocal cross-sections showing (**a**) perfusion-based delivery, (**b**) control MNs without DOX, (c) DOX-loaded MNs insertion. Quantitative cell death profiles validate MN efficacy. (**d**) Z-stacked cross-sections of melanoma with (right) and without **(left**) DOX treatment. Scale bars: 200 μm [122]. This figure is adapted from Ref. [122] under the Creative Commons Attribution 4.0 (CC BY 4.0) license.

Barros et al. (2024) and colleagues developed a high-throughput 3D bioprinted skin/skin cancer-on-a-chip model designed to overcome the limitations of traditional systems in replicating native skin architecture and enabling localized drug delivery [122]. The method starts with the creation of a microfluidic skin-on-a-chip system that includes PDMS chambers and a porous membrane, as shown in Figure 4A(a). This allows for the application of microneedles (MNs) for transdermal drug delivery and the culture of layered skin tissue. Keratinocyte differentiation is supported by the air–liquid interface (ALI) culture as shown in Figure 4A(b), which encourages stratification of the epidermis. Figure 4B–C illustrates layer formation and morphological integrity. Well-organized 3D dermal layers, as shown in Figure 4B, and the sequential development of epidermal layers on days 10, as shown in Figure 4C(a), and 14, as shown in Figure 4C(b), are seen in confocal imaging [122]. Fluorescence labeling of nuclei, actin, α-SMA, and pan-cytokeratin confirms the arrangement of cells. The dermal and epidermal layers’ time-dependent assembly and their measured thicknesses, which support full skin maturation, are further described in Figure 4D(a,b). Marker expression and tissue identity are shown in Figure 4E. Confocal pictures and measurements of the epidermal markers Keratin 14/19, as shown in Figure 4E(a), Filaggrin/Keratin 10 as shown in Figure 4E(b), and dermal markers Collagen I/Fibronectin as shown in Figure 4E(c), validate proper stratification [122]. With cornified epidermis and layered dermis, H&E staining, as shown in Figure 4E(d), verifies architectural resemblance to native skin.

The epidermis’s relative spatial expression of FLG, K14, and K10 is depicted in Figure 4F, which shows clear localization from the stratum corneum to the basale. In Figure 4G(a), a metastatic layer is integrated into the dermis to simulate melanoma. Tumor invasion towards the media channel is demonstrated by live/dead staining following a 24-h ALI culture, as shown in Figure 4G(b). Melanoma positioning is confirmed by a 3D reconstructed image, as shown in Figure 4G(c) [122]. After that, DOX-loaded MNs were created and described as shown in Figure 4H. Their application in the skin cancer model with structural views Figure 4H(a), mechanical properties Figure 4H(b,c), drug release profiles Figure 4H(d) and schematic, analysis of confocal image as shown in Figure 4H(a–g), all demonstrate successful and consistent penetration into tumor zones (~600 μm). The effectiveness of drug delivery is finally assessed in Figure 4I. A comparison of Figure 4I(a) perfusion, Figure 4I(b) DOX-free MNs, and Figure 4I(c) DOX-loaded MNs is shown in cross-sectional images. Melanoma cell death and drug localization attest to MN-based delivery’s superior performance. Z-stack views Figure 4I(d) demonstrate the targeted cytotoxicity of DOX-loaded MNs, confirming the system’s suitability for therapeutic modeling and high-throughput screening [122].

In conclusion, the advancement of high-throughput techniques for creating 3D bioprinted skin cancer organoids represents a revolutionary development in preclinical cancer research. Organoid production is significantly faster and more reproducible thanks to automation and multiplexed printing. Integration with organ-on-chip and microfluidic platforms allows for real-time drug and immune response studies as well as dynamic modeling of the tumor microenvironment. Lastly, the standardization required for clinical translation is guaranteed by strong quality control procedures and scalable systems. The implementation of bioprinted organoids in therapeutic screening and personalized diagnostics is made possible by these developments taken together.

## 5. 3D Bioprinted Organ-on-Chips for Skin Cancer Detection: A Converging Platform for Precision Diagnostics

An innovative method for simulating the human skin microenvironment for the early detection and research of skin cancers, especially melanoma and non-melanoma types, is provided by the combination of 3D bioprinting and organ-on-chip (OoC) technologies. The intricate structure, diversity, and dynamic microenvironmental interactions of human skin tumors are not adequately represented by conventional in vitro and in vivo models. On the other hand, 3D bioprinted organ-on-chip platforms offer miniature, physiologically relevant systems that can accurately replicate the biochemical and biomechanical characteristics of skin tissue. The first step in creating these hybrid systems is choosing biomimetic bioinks, which usually include hydrogels that resemble extracellular matrix (ECM) and skin cells that are either patient-derived or genetically modified, such as fibroblasts, tumor cells, melanocytes, keratinocytes, and melanocytes. These cellular components can be precisely spatially deposited to replicate the epidermal and dermal compartments using the layered 3D bioprinting process [123,124,125]. In order to replicate vascular flow, mechanical stress, and the interstitial transport of nutrients and medications, these constructs are subsequently incorporated into microfluidic chips that have perfusable channels. This platform’s capacity to replicate the tumor microenvironment, including immune cell infiltration, hypoxic gradients, and dynamic cytokine signaling, is one of its main advantages.

This makes it possible to track the beginning, spread, and invasion of cancer in real time. Additionally, the chip’s biosensors allow for the quantitative identification of tumor biomarkers like S100B, MIA, and LDH, which promotes early diagnosis [126,127,128,129,130]. These sensors measure electrical impedance, metabolic activity, and secreted biomarkers to monitor how cells react to stimuli like UV light or medicinal drugs. These platforms also facilitate high-throughput screening for immunotherapeutics and chemotherapeutics, providing information on patient-specific reactions. Individualized diagnostic models can be created using customized bioinks made from tumor cells derived from the patient. Additionally, the platform facilitates the co-culturing of immune cells, including macrophages and T cells, allowing for immunophenotyping and the investigation of immune evasion mechanisms in skin cancers. Notwithstanding the benefits, there are still issues to be resolved, such as preserving long-term cell viability, attaining high-resolution vascularization, and standardizing fabrication procedures. These restrictions are gradually being addressed, though, by recent developments in biocompatible materials, real-time imaging, and microfluidic control systems [14,131,132,133,134,135,136].

**Figure 5:**
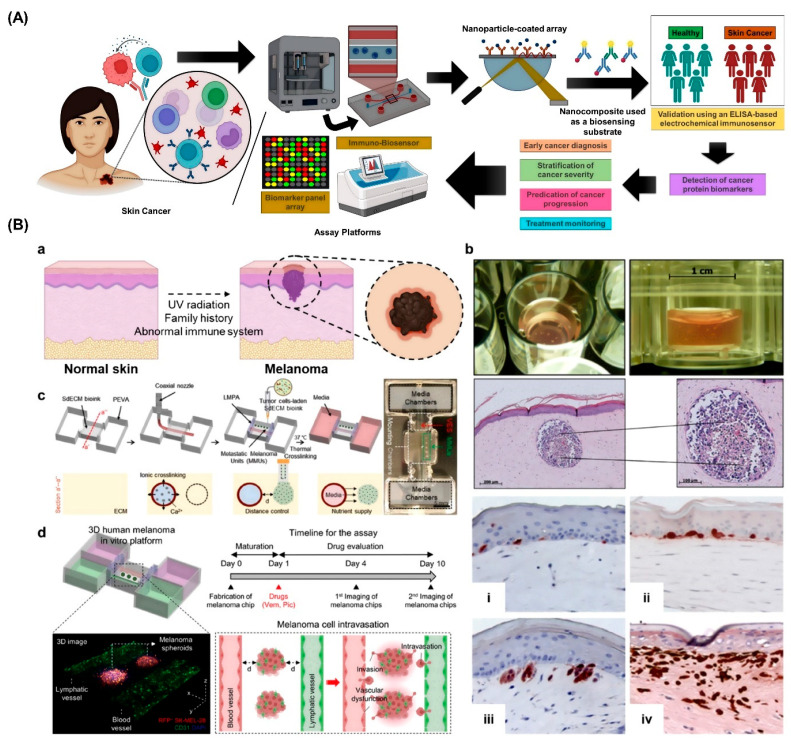
Integrated biosensing and engineered melanoma models for high-throughput cancer detection and disease modeling. (**A**) Schematic representation of a nanotechnology-based biosensing strategy for skin and breast cancer detection. The process begins with a drop of blood obtained from breast cancer (BC) patients, which is applied to a nanoparticle-coated microarray for high-throughput screening of cancer biomarkers, including those associated with skin cancer. Candidate biomarkers identified through this screening are then validated using ELISA in a larger patient cohort. This validation enables applications in early diagnosis, disease stratification, prognosis, and monitoring of treatment responses. Based on the performance of the selected biomarker panel, ELISA-based electrochemical immunosensors or biosensors may be developed for clinical applications in breast cancer detection. Created with BioRender.com (accessed on 25 July 2025). (**B**) Representative models and fabrication strategies for melanoma-skin constructs. (**a**) Major risk factors contributing to melanoma development. (**b**) Tissue-engineered approaches for constructing in vitro melanoma models, including sequential stages of disease progression: (**i**) localization of melanocytes at the dermal–epidermal junction, (**ii**) early melanoma cell clustering at the basement membrane, (**iii**) invasion of melanoma cells into the dermis, and (**iv**) aggressive dermal invasion. (**c**) In-bath bioprinting of melanoma spheroids with a perfusable vascular channel to mimic the tumor microenvironment. (**d**) In-bath bioprinting of melanoma stroma embedded with a paired blood and lymphatic vessel system for modeling tumor–vasculature interactions [137]. This figure is adapted from Ref. [137] under the Creative Commons Attribution 4.0 (CC BY 4.0) license.

Three-dimensional (3D) bioprinted organ-on-chip platforms, which combine tissue engineering, microfluidics, and nanotechnology, are becoming increasingly potent instruments for skin cancer precision diagnostics. Using nanoparticle-coated microarrays, a biosensing approach based on nanotechnology allows for the high-throughput detection of cancer biomarkers, as shown in Figure 5A. These arrays are used to find possible markers, such as those linked to skin cancer, using a tiny blood sample taken from patients with breast cancer. Promising biomarker candidates are validated by ELISA in sizable patient cohorts after array-based screening, enabling their application in prognosis, early-stage diagnosis, disease stratification, and therapeutic monitoring. After validation, these biomarkers can be incorporated into biosensor platforms or electrochemical immunosensors for clinical use, furthering the development of accurate and non-invasive cancer diagnostics. Engineered melanoma skin models that depict the various phases of melanoma invasion and are created through bioprinting techniques are shown in Figure 5B [137]. Key risk factors for the development of melanoma, including genetic predisposition and UV exposure, as shown in Figure 5B(a).

The disease progression is shown step-by-step, as shown in Figure 5B(b), starting with the presence of melanocytes at the dermal–epidermal junction and continuing through early tumor clustering, dermal infiltration, and deep tissue invasion. Skin constructs that have been tissue-engineered are used to replicate these pathological stages. Melanoma spheroids can be integrated with perfusable vascular channels using sophisticated in-bath bioprinting techniques, as shown in Figure 5B(c), which precisely replicates the tumor microenvironment. Additionally, modeling of tumor–vasculature interactions—which are essential for researching metastasis and immune evasion—is made possible by bioprinted stroma that incorporates both blood and lymphatic vessels as shown in Figure 5B(d).

When combined, these systems provide a complete and expandable platform for high-throughput drug testing, biomarker validation, and disease modeling, establishing 3D bioprinted organ-on-chips as revolutionary instruments in precision oncology [137]. In conclusion, 3D bioprinted organ-on-chip systems are an effective way to improve diagnostic accuracy and model the pathophysiology of skin cancer. These platforms open the door for individualized and non-invasive skin cancer diagnostics by fusing microfluidic dynamics with cellular complexity, which eventually leads to earlier detection and better treatment approaches.

## 6. Applications in Diagnosis and Personalized Therapy

Three-dimensional (3D) bioprinted skin cancer organoids are a revolutionary development in personalized medicine and cancer diagnosis. These engineered constructs provide previously unheard-of possibilities for high-throughput drug screening, genomic profiling, and immunotherapeutic evaluation by simulating the intricate architecture and pathophysiological microenvironment of human tumors. The translational gap between bench research and clinical application is filled by 3D bioprinted organoids, which overcome the shortcomings of conventional 2D cultures and animal models, especially in capturing tumor heterogeneity and patient-specific responses. The use of skin cancer organoids in drug screening and sensitivity testing is among their most prominent uses. The spatial and cellular complexity of human tumors is frequently not replicated by traditional monolayer cultures, which leads to inaccurate efficacy data. However, within a biomimetic extracellular matrix, bioprinted organoids can incorporate a variety of cell types, such as immune cells, fibroblasts, endothelial cells, and tumor cells [138,139,140].

This makes it possible to summarize the kinetics of drug penetration and tumor-stroma interactions. These organoids allow hundreds of drug candidates to be tested simultaneously across a wide panel of patient-derived samples when used in high-throughput formats. For example, certain kinase inhibitors that specifically target oncogenic signaling in drug-resistant subtypes have been found using organoids from melanoma and squamous cell carcinoma. Furthermore, fluorescent or imaging-based assays can be used to track dynamic responses in real time, such as invasion suppression, proliferation inhibition, or apoptosis induction. By identifying the most effective therapeutic agents for each patient, this individualized screening method shortens the time to treatment and enhances clinical results. 3D bioprinted organoids are useful tools for genomic validation and molecular profiling in addition to pharmacological testing. The epigenetic signatures and mutational landscape of the original tumors, such as p53, NRAS, BRAF, and other oncogenic drivers frequently found in skin cancers, are preserved in patient-derived organoids [141,142,143,144].

Researchers can link genomic changes to treatment response by using high-throughput sequencing and transcriptomic analysis on organoid tissues. Finding biomarkers and predicting resistance mechanisms are improved by this combination of omics data and functional assays. To help with the logical design of combination therapies, gene expression profiling of skin cancer organoids undergoing targeted therapy, for instance, can identify compensatory pathways that mediate drug resistance. Additionally, functional interrogation of particular genes is made possible by the use of CRISPR-Cas9 genome editing within organoids, confirming their roles in drug response or tumor progression. When combined with the structural accuracy of bioprinted models, these genomic tools help to improve precision oncology by guiding clinical judgment and medication development. Immunotherapy has transformed the treatment of skin cancer, especially melanoma, but it is still very difficult to predict how a patient will react. Organoids made from bioprinted patients present a viable way to assess immunotherapy [145,146,147].

Researchers can replicate immune-tumor interactions in a controlled ex vivo setting by integrating autologous immune cells, such as peripheral blood mononuclear cells (PBMCs) or tumor-infiltrating lymphocytes (TILs), into the bioprinted constructs. This makes it possible to test adoptive cell transfer procedures, cytokine treatments, or immune checkpoint inhibitors in a customized setting. T-cell activation, cytotoxic tumor cell death, and PD-1/PD-L1 signaling can all be replicated by immune-active organoids, according to recent research. These systems also make it possible to assess the immune evasion tactics used by tumor cells, such as the release of inhibitory cytokines or the expression of immune-suppressive ligands. As a result, organoid-based immunoassays can be used as predictive instruments for patient stratification, allowing medical professionals to determine who will respond and who will not before starting treatment [148,149,150]. In conclusion, the high-throughput 3D bioprinted skin cancer organoids offer a thorough and physiologically appropriate platform for improving therapy customization and diagnosis. Through molecular profiling, immunotherapy evaluation, and drug screening, these models aid in the creation of customized treatment plans that are less harmful and more effective. The incorporation of organoids into clinical workflows has enormous potential to revolutionize cancer treatment in the future as bioprinting technologies and biomaterials advance.

## 7. Future Perspectives

The creation of skin cancer organoids using high-throughput 3D bioprinting technology has created new opportunities for tumor complexity modeling, improving diagnostic precision, and allowing for individualized treatment plans. With the potential to completely transform how physicians approach treatment planning, drug sensitivity profiling, and immunotherapy response prediction, the field is moving closer to clinically meaningful applications. However, several crucial translational and regulatory issues need to be resolved to move from experimental research to standard clinical practice. Verifying that bioprinted skin cancer organoids can replicate the physiological and pathological features of native tumors, such as their cellular structure, genetic mutations, and microenvironmental interactions, is essential for clinical translation [151,152,153].

To guarantee reproducibility and comparability of results across labs, standardization of procedures across bioprinting platforms, bioink compositions, and cell sources is crucial. Furthermore, before approving these organoid models for clinical decision-making or drug screening, regulatory bodies like the FDA and EMA demand comprehensive validation studies that show their safety, effectiveness, and dependability. With informed consent, data security, and biobanking procedures in place, ethical issues of the use of patient-derived cells must also be properly handled. The widespread use of 3D bioprinted organoid technology is still constrained by many issues, despite encouraging developments. The intricacy of simulating the tumor microenvironment, which includes stromal interactions, immune cell infiltration, and vasculature—all of which are important factors in the development of cancer and resistance to treatment—is one of the main challenges. The creation of generalized models is made more difficult by the diverse nature of skin cancers, including melanoma, squamous cell carcinoma, and basal cell carcinoma. Reduced biological functionality or structural resolution is frequently the price paid for high-throughput fabrication. Furthermore, cross-study comparisons are challenging due to the absence of benchmarking tools and standardized assays among research groups [99,154,155]. Table 3 shows a comparative overview of advanced 3D in vitro skin models designed to mimic physiological architecture, cellular heterogeneity, and immune–tumor interactions. It highlights their relevance for studying melanoma progression, therapeutic response, and microenvironmental dynamics, offering a concise reference for model selection.

**Table 3:** Summary of selected 3D in vitro systems—including organoids, human planar skin constructs, and microfluidic platforms—utilized to replicate various features of skin physiology, structural organization, and the melanoma tumor microenvironment, highlighting the incorporation of immune components.

Model Type	Method	Cell Composition	Matrix Used	Purpose	Limitations	Ref.
Spherical Melanoma Organoids	Co-culture with fibroblasts	Human skin fibroblasts; melanoma cell lines (WM1366/1205Lu)	Bovine Collagen I	Explore stromal influence on tumor development and resistance	Limited to a single healthy cell type; lacks layered skin structure	[156]
	Co-culture with endothelial cells	HUVECs; melanoma cell lines (A375/M21)	-	Investigate tumor angiogenesis	Absence of healthy skin cells; non-functional capillary networks	[157]
	Three-cell model	Fibroblasts (CCD-1137Sk), keratinocytes (HaCaT), melanoma cells (SK-MEL-28)	Endogenous Collagen IV	Model early-stage melanoma, assess chemotherapy response	No cornified epidermal layer formed	[158]
	Five-cell model	Primary fibroblasts, keratinocytes, melanocytes, adipocytes; melanoma cells (SK-MEL-28)	-	Tumor-stroma crosstalk in melanoma	Does not replicate melanoma penetration	Unpublished
Immune-Competent Melanoma Organoids	Air-liquid interface culture	Stromal and immune cells from tumor biopsies	Type I Collagen	Personalized immunotherapy	No healthy skin or epidermal stratification	[159,160,161]
	Combined lymph node model	Melanoma tissue; lymph node-derived immune cells	Hyaluronic acid/Collagen hydrogel	Personalized treatment screening	Few patient samples; lacks full skin context	[162]
	Autologous lymphocyte co-culture	Melanoma tissue; peripheral lymphocytes	Matrigel	Candidate selection for immunotherapy	Limited patient scope; lacks full skin structure	[163]
Melanoma on Planar Skin Constructs	Spheroid/Cell injection	Keratinocytes, fibroblasts; melanoma cell lines (WM35, SK-MEL-28, SBCL2, etc.)	DED, Collagen I, Alvetex scaffold	Study melanoma invasion and drug responses	Missing cell types like melanocytes, vasculature	[164,165,166]
	Vascularized melanoma model	HMVECs, keratinocytes, fibroblasts; melanoma lines	-	Drug screening in vascularized environment	Time-consuming; low throughput	[167]
Immunocompetent Planar Models	Activated immune cell addition	Keratinocytes; CD4+ T cells or Langerhans cells	DED, Collagen I	Psoriasis, allergy, drug testing	Donor mismatch and limited skin cell diversity	[168,169,170,171]
	Bioprinted with macrophages	Keratinocytes, fibroblasts, macrophages (M1/M2)	Custom bioink with nanofibrillated cellulose, fibrinogen, etc.	Chronic inflammation (e.g., atopic dermatitis)	Lacks melanocytes, vasculature	[172]
	Bioprinted wound models	Keratinocytes, fibroblasts, HUVECs, macrophages	Collagen I + plasma-based fibrin bioink	Wound healing and inflammation	Missing melanocytes	[173]
Immune-Competent Skin Constructs with Melanoma	Co-culture with melanoma and immune cells	Keratinocytes, fibroblasts, melanocytes, melanoma cells, dendritic or T cells	Collagen I, DED	Tumor-immune interaction, progression, and immunotherapy	No vasculature; no leukocyte extravasation	[174,175,176,177]
Melanoma-on-a-Chip Systems	Microfluidic integration	Keratinocytes, fibroblasts, melanoma cells (WM-115)	Collagen	Cell-cell crosstalk studies	No stratified skin architecture	[178]
	Skin-on-chip with immune components	HaCaT, U937 or HL-60 cells, HUVECs	Collagen I	Allergy and immune response modeling	Lacks full dermal/immune composition	[179,180,181]
	Melanoma-immune chip systems	Melanoma spheroids, immune cells from biopsy	Collagen I	Immunotherapy and drug screening	No healthy skin structure; immune cell recruitment limited	[182,183]
	Vascularized chip with immune cells	HUVECs, melanoma cells (BLM), whole blood	Gelatin	Inflammation modeling	No skin layers included	[184]
	Circulating melanoma-neutrophil interactions	Melanoma A-375/A-375 MA2, neutrophils	Fibrin	Tumor cell extravasation, metastasis	Not representative of skin architecture	[185]

Using microfluidic systems, sometimes referred to as organ-on-chip platforms, in conjunction with bioprinted constructs to more accurately replicate physiological perfusion and dynamic nutrient exchange is one of the emerging solutions to these problems. Replicating native tissue complexity through co-culturing cancer cells with fibroblasts, endothelial cells, and immune cells in bioengineered matrices is also showing promise. More biocompatible and adjustable hydrogels that sustain long-term organoid growth while preserving cellular viability and phenotype have been developed as a result of advancements in biomaterial science [113,186,187]. Furthermore, to improve throughput and analytical accuracy, organoid-based screening platforms are integrating artificial intelligence (AI)-driven imaging and data analysis pipelines. In order to facilitate customized therapy testing, future developments in precision skin cancer modeling are probably going to concentrate on customizing organoid systems with patient-derived cells. Combining bioprinted organoid responses with genomic and transcriptomic data will aid in improving drug toxicity and efficacy prediction algorithms. To satisfy the needs of clinical labs and the pharmaceutical sector, these platforms’ scalability must also be considered. To study oncogenic drivers and therapeutic targets in a controlled environment, researchers are also investigating the use of gene-editing technologies, such as CRISPR/Cas9, to introduce or correct mutations in bioprinted organoids [188,189,190].

Integrating multi-omic profiling—including single-cell RNA sequencing (scRNA-seq), spatial transcriptomics, and phospho-proteomics—provides a powerful framework to enhance the molecular and functional resolution of bioprinted organoids. These approaches enable detaile-d characterization of cellular heterogeneity, dynamic signaling networks, and complex tumor–microenvironment interactions, which are essential for understanding disease mechanisms and tailoring personalized therapies. When coupled with artificial intelligence-driven drug-response prediction models, such comprehensive datasets facilitate high-throughput screening and the rational identification of patient-specific therapeutic strategies. By bridging advanced omics technologies with 3D bioprinted tumor platforms, this integrative approach not only improves mechanistic insights but also substantially increases the translational potential of organoid-based models for precision oncology and clinical decision-making [83,191,192,193,194].

Bioprinted organoid models have emerged as highly predictive platforms for evaluating clinical drug responses, facilitating high-throughput screening and the development of personalized therapeutic strategies. By closely recapitulating patient-specific tumor architecture and microenvironmental features, these models accelerate preclinical testing while reducing dependence on animal models, thereby improving the efficiency of drug design and efficacy assessment. To maximize translational potential, this review addresses regulatory considerations, including FDA and EMA guidelines, and highlights the implementation of robust quality-control frameworks. Emphasizing reproducibility, safety, and standardization, these strategies collectively support the integration of bioprinted organoids into clinical pipelines, underscoring their promise as reliable tools for precision oncology and personalized medicine [195,196,197,198].

Collaboration between academic institutions, regulatory agencies, and biotechnology companies is crucial to ensuring broad adoption and utility. Additionally, creating centralized databases, creating open-access organoid biobanks, and investing in staff training will hasten innovation and interdisciplinary knowledge exchange. In summary, high-throughput 3D bioprinted skin cancer organoids offer a platform that connects fundamental research and clinical use, marking a revolutionary development in the field of oncology. Even though this technology has obstacles to overcome, especially in the areas of biological complexity, reproducibility, and regulation, its future looks bright. Bioprinted organoids have the potential to become a crucial part of precision medicine by overcoming present obstacles and embracing new technologies and interdisciplinary partnerships. This will allow for more efficient, individualized, and timely treatments for patients with skin cancer.

## 8. Conclusions

High-throughput 3D bioprinting of skin cancer organoids is an innovative development in cancer modeling, diagnosis, and personalized therapy. They allow the reconstruction of complex tumor microenvironments, including fibroblasts, keratinocytes, endothelial cells, and immune cells. Organoids are similar to traditional 2D cultures or animal models in that they closely mimic the histological architecture, genetic mutations, and immune responses of native skin tumors, including melanoma and non-melanoma. This enables drug screening, immunotherapy assessment, and treatment planning with a focus on specific drugs and immunotherapy. However, they must address a number of translational and regulatory hurdles, even though they promise. For clinical use, organoids must replicate physiological tumor features and be validated on a variety of platforms. The standardization of bioprinting techniques, cell sources, and bioink compositions is important for reproducibility and comparability of results across laboratories. Regulatory bodies like the FDA and EMA need to validate these models extensively before they can be used in clinical workflows. Replicating complete tumor microenvironment complexity, including vasculature, stromal interactions, and immune infiltration, remains challenging. Many models still lack the full spectrum of skin architecture and cell diversity. These constraints are being overcome by innovations such as organ-on-chip systems, improved biomaterials, and improved analysis pipelines with artificial intelligence. Additionally, CRISPR/Cas9 gene editing is being used to allow personalized organoid systems to predict therapeutic outcomes by patient-derived cells combined with CRISPR/Cas9 gene editing. 3D bioprinted skin cancer organoids provide a promising platform for integrating basic research and clinical applications. While there are still limitations on biological complexity, standardization, and regulatory approval, technological advances and collaboration are enabling them to be more widely implemented. As further refinements occur, bioprinted organoids could become a central part of precision oncology, allowing more accurate, individualized, and effective treatments for skin cancer patients.

## Data Availability

No new data were created or analyzed in this study.
